# Fatigue Performance of Type I and Type II Fibre Bragg Gratings Fabricated by Femtosecond Laser Inscription through the Coating

**DOI:** 10.3390/s22228812

**Published:** 2022-11-15

**Authors:** Naizhong Zhang, Suzana Turk, Claire Davis, Wing K. Chiu, Tommy Boilard, Martin Bernier

**Affiliations:** 1 Department of Mechanical and Aerospace Engineering, Monash University, Wellington Rd, Melbourne, VIC 3800, Australia; 2 Defence Science and Technology Group, 506 Lorimer Street, Fishermans Bend, Melbourne, VIC 3207, Australia; 3 Centre d’Optique, Photonique et Laser (COPL), Université Laval, Québec, QC G1V 0A6, Canada

**Keywords:** fibre Bragg gratings, trans-jacket inscription, FBG fatigue testing, femtosecond inscription, Type I and Type II FBGs

## Abstract

Strain sensing technology using fibre Bragg grating (FBG) sensors is an attractive capability for aerospace structural health monitoring (SHM) and assessment because they offer resistance to harsh environments, low maintenance, and potential for high density and high strain sensing. The development of FBG inscription techniques through the fibre polymer coating using infrared (IR) lasers has overcome the mechanical weaknesses introduced by removal of the fibre coating, which is typically required for conventional UV laser inscription of FBGs. Type I and Type II femtosecond gratings are fabricated using through-coating inscription techniques, but the higher laser energy used for Type II gratings damages the glass fibre core, impacting mechanical performance. This paper investigates the fatigue performance of Type I and Type II through-coating FBG sensors with different fibre geometries and photosensitisation approaches to evaluate their overall reliability and durability, with a view to assess their performance for potential use in civil and defence SHM applications. The fatigue performance of FBG sensors was assessed under high-strain and high-frequency mechanical loading conditions by using a custom-designed electro-dynamically actuated loading assembly. In addition, pre- and post-fatigue microscopic analyses and high-resolution reflection spectrum characterisation were conducted to investigate the failure regions of the fibres and the effect of fatigue loading on reflection spectrum features. As expected, Type I gratings had a significantly higher fatigue life compared to Type II gratings. However, Type II gratings performed significantly better than conventional UV laser-inscribed FBGs and electrical foil strain gauges. Type II gratings withstand higher temperatures, and are therefore more suitable for application in harsh environments.

## 1. Introduction

Fibre Bragg gratings (FBGs) have been broadly applied as sensors for strain, temperature, pressure, and vibration measurements [1,2,3] since their first demonstration in this capacity over 30 years ago [4]. The most significant advantage of the FBG sensor is the built-in self-referencing capability. The fibre itself is a sensor, as well as a signal transducer. As the wavelength-encoded information given by the Bragg grating is an absolute parameter, the output signal is immune to power fluctuation, optical fibre bend loss, and connection loss along the signal path. Wavelength-division-multiplexing (WDM) is a fibre optic technology that multiplexes a series of signals onto a single optical fibre by using different wavelengths of the light source. FBGs detect measurands at multiple points along a sensor line by using WDM and transmit the signal over a long distance with low power loss [5], enabling quasi-distributed and distributed sensing applications. Due to their small physical footprint, corrosion resistance, and immunity to electromagnetic interference, FBG sensors are well suited for integrated distributed sensing for long-term strain, vibration, and temperature measurement to inform aircraft structural health monitoring (SHM) [6,7]. They can be embedded or surface mounted onto host structures and permit in situ SHM to be undertaken on aircraft [8]. Richards et al. [9] used approximately 3000 FBG strain sensors to monitor the in-flight wing shape of the NASA Ikhana unmanned aerial vehicle. The strain results obtained from the FBGs and the electrical foil gauges bonded to the upper wing surface were in agreement. Nicolas et al. [10] studied 778 FBG sensors with a spacing of approximately 12.5 mm on a three-tier whiffletree system used to simulate an in-flight lift distribution on the wing structure. The maximum strain measured during the test was 2000 µε, with strain measured by FBG sensors and strain gauges in agreement. Moreover, the FBG sensor arrays provided a higher spatial density of strain measurements than was practically achievable with conventional electrical resistance foil strain gauges (FSGs) to present more information on the mechanical performance of the structure under load.

The reliability and durability of strain sensing systems are crucial for application in aircraft SHM. It was reported in [11] that standard commercially available FSGs were not durable, required replacement midway through the test, and introduced significant cabling weight that influenced the results of military aircraft fatigue testing. The researchers considered using FBG strain sensors to replace the less durable FSGs in the aircraft structure fatigue test. However, the mechanical strength and fatigue life of FBG sensors require comprehensive evaluation before they are transitioned to military aircraft SHM applications. The reported variability of FBG mechanical behaviour under relatively high amplitude cyclic loading is primarily dependent on the inscription techniques used to create the sensors [12].

FBG inscription is typically achieved using UV radiation [13,14] or infrared (IR) femtosecond (fs) laser pulses [15,16]. UV-induced refractive index changes rely on single- and multi-photon absorption of hydrogen-loaded fibres, which enhance inscription photosensitivity [17,18,19,20]. The environmental protection coating applied to optical fibres is typically not transmissive in the UV region of the electromagnetic spectrum, and therefore must be removed prior to inscription. The standard FBG fabrication technique was stripping and recoating. Stripping and recoating is undesirable because it is time and labour intensive and mechanically damages the glass fibre surface, thereby reducing mechanical performance and fatigue life.

Ang et al. [20] reported that the fatigue failure strain of typical stripped and recoated FBGs was approximately 5000 µε, which was only 10% of the tensile strain limit of the pristine fibre. Yoon [21] reported that mechanical damage to the fibre surface caused by stripping significantly reduced failure stress. New approaches have been developed to achieve fibre coatings that are partially UV-transparent [22], and UV-FBGs have been written through the coating of an enhanced photosensitivity fibre [23]. A near-UV source was used to reduce fibre coating absorption to 25%. However, UV irradiation through the coating has not yet achieved index modulation levels comparable to those in conventionally-written FBGs in silica fibres, which is a potential disadvantage. In addition, photon-induced index changes of Type I-UV gratings caused by densification-induced compaction of the core network are erased at elevated temperatures [24], thus limiting the operating temperature range of UV-FBGs.

The draw tower grating (DTG) technique inscribes the grating into the fibre core prior to the application of a protective polymer coating using an automated fabrication process. In 1994, Askins et al. [25] demonstrated the fabrication of reflective Bragg gratings in line during the fibre draw process with a single pulse of an excimer laser. Although the fibre mechanical strength can be essentially preserved using this methodology [26], the spectral properties of the DTG are significantly diminished due to the single-pulse exposure during the draw process, which limits the sensor performance [27,28].

Pulsed IR-induced modification of the refractive index relies on a multi-photon absorption process [16,29]. In the early 2000s, fs-IR pulses were developed to inscribe FBGs in bulk silica fibres or Ge-doped fibres using both the phase mask [30] and point-by-point (PbP) inscription techniques [29]. PbP inscription was reported to reduce the mean failure stress of the fibre by over 80%, since it relies on the formation of void-like defects [31]. In comparison, the phase mask technique was reported to reduce the mean failure stress by only 15–25% [32]. The fs-IR laser-pulsed FBG inscription technique has some significant advantages:

1. Fs-IR pulses facilitate writing through the coating because most polymers are IR transparent [33,34]. The FBGs written through the coating are also known as trans-jacket or trans-coating FBGs. With no requirement to strip the fibre before laser exposure, the manufacturing process is much simpler and lends itself more readily to automation, reducing unit cost and increasing sensor reliability and durability.
2. Fs-IR pulses also make writing in non-UV photosensitive glasses possible. For example, FBGs can be written in all-silica core fibres with both UV and IR light [34,35], but mainly with IR fs pulses for fluoride [36] glass fibres.
3. Type II gratings inscribed using fs-IR pulses have similar properties to Type II UV-inscribed gratings regarding thermal stability, but much higher index modulation and superior spectral quality [16].
4. High index modulation can be achieved by IR laser exposure without special photosensitisation of the fibre, such as hydrogen loading or special core doping with photosensitive materials.

Previous work reported by the authors comparatively evaluated the fatigue performance of a series of Type I FBGs fabricated using different inscription methodologies [37]. The published results showed that trans-jacket FBG sensors manufactured with fs-IR pulses and a phase mask [38] demonstrated superior fatigue performance compared with commercially supplied, stripped, and recoated FBGs. A Ti-sapphire regenerative amplifier system that produces 3.5 mJ energy pulses at 1 kHz with a central wavelength of *λ* = 806 nm was used. The temporal width of the Fourier transform-limited pulses was measured to be ~34 fs. The beam was directly focused using an acylindrical lens with a focal length of 8 mm through a uniform silica phase mask onto the fibre. The focussing lens is mounted in a piezoelectric stage to sweep the focal line during inscription to ensure that the writing beam is overlapping the entire fibre core [39].

Many studies have reported the stability of spectral responses of Type II trans-jacket gratings at high temperatures and their excellent annealing behaviour [40,41,42,43]. However, it was reported in [43,44] that the silica core of Type II fs-IR gratings was damaged by the inscription process. Habel et al. [44] conducted pull-tests on both fs-IR laser-inscribed Type I and Type II trans-jacket FBGs. The results showed that polyimide-coated silica fibres containing Type I FBGs had a similar mean failure stress compared with the pristine fibres. It indicated that the Type I trans-jacket active core alignment inscription technique did not degrade the mechanical strength of the pristine fibre. Pull-tests conducted on Type II gratings revealed a mean breaking stress of 0.5 GPa, ten-fold below the 5.3 GPa value obtained for pristine fibres and Type I gratings. High-temperature hydrogen loading, which is used to increase the photosensitivity of glass fibres, also harms fibre tensile strength [45]. The long-term reliability and durability of trans-jacket FBG sensors remains to be fully characterised.

Few studies have systematically investigated the mechanical strength of Type I and II trans-jacket FBGs using IR inscription, and no studies have comprehensively validated the fatigue performance of Type II trans-jacket FBG sensors under high cyclic load at high frequency. The ultimate goal is to facilitate enhanced strain sensing performance for SHM of civil and military aerospace platforms in harsh operational environments. The present experimental study examines and compares the fatigue performance of Type I and Type II fs-IR trans-jacket FBGs by conducting fatigue tests using a custom-designed assembly. The fatigue life of FBG sensors was characterised under tensile fatigue loading at peak strains up to 15,000 µε at a cycling frequency of 100 Hz. The fibre failure mechanism was determined using microscopy and spectrum characterisation results. The outcome of this study will contribute to a broader research program qualifying trans-jacket FBG strain sensors for use on aircraft structures.

### 1.1. FBG Working Principles

A FBG is a periodic modulation of the refractive index that can be written in the optical fibre core using mainly UV [13,14] and IR laser sources [15,16]. The Bragg wavelength, or resonance condition, is expressed by:

(1) $$\lambda_{B}=2n\Lambda$$

where Λ is the grating pitch and *n* is the effective refractive index of the fibre core. A narrowband spectral component at the Bragg wavelength $\lambda_{B}$ is reflected when a broadband source of light is injected into the fibre. The shift of the Bragg wavelength in the reflection spectrum with strain and temperature can be approximated as:

(2) $$\frac{\Delta \lambda_{B}}{\lambda_{B}}=\left(\alpha +\xi \right)\Delta T+\left(1-\rho_{e}\right)\varepsilon$$

where ε is the longitudinal strain experienced by the FBG, ΔT is the external temperature variation at the FBG location, α is the linear coefficient of thermal expansion, *ξ* is the thermo-optic coefficient, and $\rho_{e}$ is the effective photo-elastic constant of the fibre core material [28]. Typical strain and temperature sensitivities of FBG sensors are approximately 1.2 pm/µstrain (µε) and 10 pm/°C, respectively [46]. In addition, the normalised thermal responsivity at constant strain or the measured strain response at constant temperature can be realised by several discrimination techniques [47,48,49].

### 1.2. Type I and Type II Characterisations of IR Laser-Induced FBG Sensors

The index change associated with Type I FBGs typically results from a combination of glass densification and colour-centre formation [42]. The index change in a Type II FBG results from a damaging process in the glass matrix [28]. The mechanism for achieving a refractive index change significantly differs depending on the wavelength of the light. UV-induced refractive index changes depend on colour-centre formation only, while IR-induced refractive index changes are associated with a non-linear multiphoton ionization process, bringing about material compaction or defect formation. The material response depends on the intensity of the laser exposure [50,51,52,53]. Refractive index changes at lower laser intensities (between 2 × 10^13^ W/cm^2^ and 8 × 10^13^ W/cm^2^) can be attributed to densification and colour-centre formation. Such index changes are referred to as Type I-IR gratings. At intensities above 8 × 10^13^ W/cm^2^, the glass turns birefringent due to the formation of nano-gratings. Such gratings are called Type II gratings or damaged gratings, because the modifications are above the damage threshold of the glass. A Type II-IR grating index change is of the order of Δ*n*~10^−3^, which is significantly higher than the UV radiation-induced index contrast (Δ*n*~10^−4^) [54]. Confined micro-explosions occur when intensities are higher than 3 × 10^14^ W/cm^2^, resulting in micro-voids [55]. Such micro-voids can provide extreme index contrasts of the order of Δ*n*~10^−1^. 

The durability of the Type I index change largely depends on the operating temperature. Most of the UV-induced refractive index changes are completely erased at a temperature above 450 °C. UV laser-induced draw tower gratings are erased at 250 °C [39]. Type I gratings are generally unstable at higher temperatures. Type II gratings inscribed with a single high-power pulse can remain operable at higher temperatures, but at the expense of inducing damage tracks in the fibre core [56]. The IR-induced index change in the Type II regime results from multiphoton and avalanche ionization causing plasma formation [55]. Thus, Type II damage does not anneal out at 900 °C. The micro-void structure resulting from a point-by-point inscription method provides thermal stability up to 1000 °C in standard telecom fibres [41]. Hence, Type II gratings tolerate much higher temperatures than Type I gratings, and are often adopted in elevated temperature sensing environments [57]. However, as the glass fibre is permanently damaged due to defect formation, such as micro-voids at the region of Type II gratings [42], the mechanical robustness and fatigue performance of fs-IR-induced Type II FBGs is expected to be lower than that of Type I gratings. This is reinforced by previous research that showed that the primary failure mechanism associated with fibre breakage is crack propagation from a stress concentration point (e.g., a micro-void) in the damaged glass [58].

## 2. Experimental Methods

**Figure 1:**
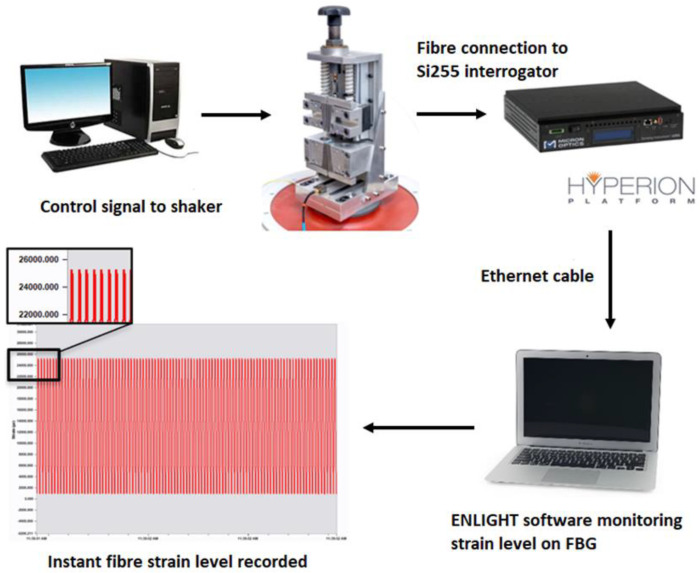
Experimental arrangement for fatigue cycling of fibre optic sensors.

Tension is considered the most damaging load case because of the relatively poor fracture resistance of optical fibres under the crack opening (mode I) condition. In the present stduy, tensile cyclic loads are applied to FBGs in coated optical fibres via a custom-designed fibre fatigue test assembly, as shown in Figure 1. Sinusoidal cyclic loading was applied using a high-capacity electrodynamic shaker (TIRA GmbH S50350, Schalkau, Germany) driven by a vibration controller. The amplitude and frequency of the assembly are adjustable and can achieve a maximum strain and frequency of 36,000 µε and 100 Hz, respectively. The induced strain on the FBG was continuously recorded using an industrial-grade optical sensing interrogator (Micron Optics Si255, Atlanta, GA, USA). This interrogator featured a dynamic strain range of approximately 130,000 µε across a wavelength range from 1460–1620 nm. The unstrained Bragg wavelength for the FBGs tested was between 1530–1550 nm, and the anticipated strain under peak loading was confirmed to be well within the interrogator sensing range. The details of the assembly design and the system optimisation were previously reported [37].

The temperature in the mechanical test laboratory where fatigue testing was carried-out was 20 ± 5 °C, which corresponds to a ±50 pm wavelength shift. The temperature-induced strain error was insignificant compared to the applied strain of 36,000 µε. Both Type I and Type II trans-jacket FBGs fabricated using a customised fs-IR grating inscription technique [38] were considered in this experimental study.

### 2.1. Microscopic Analysis of the Fibre Surface

In a previously reported experimental study [37], FBGs fabricated using the conventional strip and recoat method were found to exhibit surface flaws on the fibre coating when observed at 200× magnification using an optical microscope. Those observations were in agreement with other studies [20] which showed FBGs inscribed using the strip and recoat technique are highly likely to be damaged by the mechanical stripping method. In contrast, there was no surface degradation expected on the trans-jacket FBG fibre coating, because most of the fibre coating materials, including polyimides, are transparent to IR lasers. Type II gratings were expected to break in the active region of the sensor, since the damage writing process introduces defects in the core glass. Microscopic inspection of failure locations was carried out using an optical microscope with magnifications from 200× to 800× to investigate the failure mechanism of the trans-jacket FBGs. 

### 2.2. Spectral Analysis of FBG Reflective and Transmissive Properties

The optical reflection and transmission characteristics of FBG sensors are mainly determined by grating length, index contrast, and grating pitch, which contribute to determining their spectral profile [59]. A 1 pm high-resolution laser (Yenista TS100, EXFO, Quebec, QC, Canada) combined with a component tester (Yenista, CT400, EXFO, Quebec, QC, Canada) characterised the reflection and transmission spectra of the FBGs before and after their fatigue testing. These scans allow the critical features in the FBG spectra to be characterised, such as Bragg (centre) wavelength, side lobes, and reflectivity. The high optical power and wavelength resolution also allow accurate determination of any changes to the post-fatigue spectral profile, indicating material or coating damage. If fibres fail at the Bragg grating region during fatigue testing, the post-fatigue reflection profile will exhibit a drastic change due to damage to the grating. The pre- and post-fatigue reflection spectrum comparison and the post-failure microscopic analysis both contributed to the performance assessment of the trans-jacket FBGs.

### 2.3. Test Matrix and Fatigue Load

Single mode fibres of different dopant concentrations and core geometry were provided by the commercial suppliers OFS and FibreCore, as outlined in Table 1; 80 Type I and 80 Type II trans-jacket FBG sensors were fatigue tested with half of the FBG samples loaded with deuterium to increase photosensitivity prior to inscription. The refractive index modulation induced by 806 nm femto-second pulses was in excess of 1 × 10^−3^. The FBG reflectivity was in the range of 80–90% with a peak reflectivity of 99.9% at a wavelength of 1549 nm. The FBG grating length was 5 mm [38,39].

**Figure 2:**
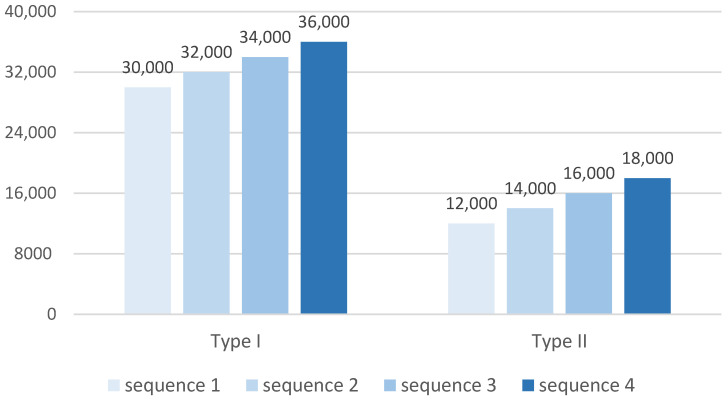
Loading regime for Type I and Type II gratings.

A stepped increasing load sequence was applied to determine an appropriate strain range for application of the sinusoidal load for each class of grating. For example, as shown in Table 2, the first segment of loading is the oscillation from 0 to 30,000 µε at a frequency of 100 Hz. Each segment contains 0.5 million loading cycles in order to ensure an adequate fatigue life is tested in each load segment, and to maintain a reasonable testing speed for the entire program. Each segment incrementally raised the peak strain by 2000 µε. This process continues until fibre failure occurs or completion of the schedule. In a preliminary test using a limited sample set, the Type I trans-jacket FBGs were found to withstand cyclic loading up to 36,000 µε (the maximum achievable by the loading apparatus) without failure. Therefore, a stepwise load test, containing a maximum peak strain of 36,000 µε, was formulated for the Type I gratings. The preliminary tests showed that Type II gratings fracture at a mean strain of approximately 17,000 µε. Thus, the loading regime was accordingly adjusted to suit the expected failure range, as shown in Table 2 and Figure 2.

A two-sample *t*-test was conducted to compare the fatigue performance of the fibres with different parameters using the recorded fatigue failure strains. This statistical test is appropriate since the population mean fatigue failure strains and corresponding standard deviations of different fibre categories are not known, and the failure strains of different fibre sample sets are independent. In addition, we assume that the failure strains of each fibre sample set are normally distributed, since a standard inscription process was applied during FBG fabrication. Our null hypothesis stated that the unknown population mean failure strains for fibres with different core geometries and those fibres with and without deuterium loading were equal. For a 99.9% confidence level (t values beyond 5.041), the null hypothesis of equal mean is rejected; thereby, the differences between the tested samples are considered statistically significant.

## 3. Results and Discussion

Table 3 shows the fatigue results for all the Type I gratings. Each Type I trans-jacket FBG sensor survived the entire test regime, accumulating 2 million load cycle applications. The results indicate that the Type I trans-jacket gratings have a high ultimate strain and fatigue performance that would be well suited for application in mechanically harsh environments that do not require operation at elevated temperatures. 

**Figure 3:**
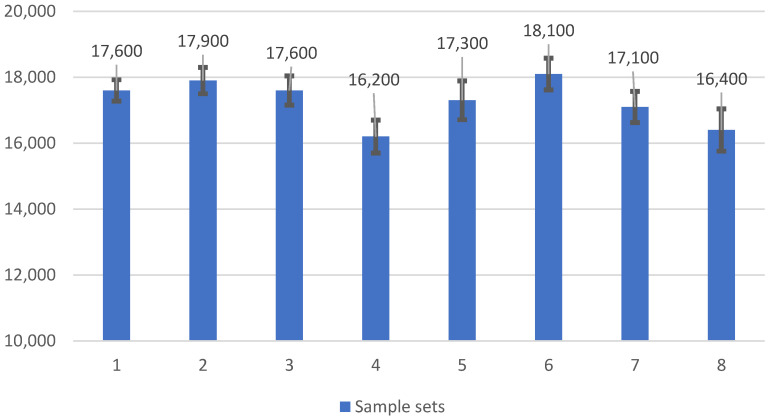
Type II trans-jacket FBG fatigue test results. The sample set numbers in Figure 3 correspond to the sample set numbers and fibre types in Table 4.

The average fatigue failure strain of Type II trans-jacket gratings was approximately 17,000 µε (Table 4). Figure 3 shows relatively small variations in fatigue failure strain and standard deviation across the test matrix. Optical fibre geometry and deuterium loading did not significantly affect fatigue life and failure strain. The fatigue failure strain of Type II FBG sensors was much lower than that of Type I. Nevertheless, the mean failure strain of Type II FBG sensors was significantly higher than typical operational strains seen in aerospace structural applications, and thus Type II gratings may be considered appropriate for applications which require extended operation at temperatures above 400 °C. The results were also considered against the reported fatigue performance of electrical resistance foil gauges, which are currently the industry standard. HBM fatigue-resistant electrical strain gauges (M-series) were reported to survive 100 million load cycles at 1000 µε strain. However, exposure to a strain range of 5200 µε curtailed their fatigue life to 1000 cycles, and they could only withstand approximately 100 load cycles at a strain range of 7000 µε [60]. Type II FBG sensors still exhibited far better fatigue life than specialty fatigue-resistant electrical gauges. Thus, in strain monitoring applications where high strain rates and high-frequency loading are required, trans-jacket-inscribed FBG sensors are likely to be more suitable than electrical resistance foil gauges.

In summary, the dominant factor in fibre fatigue performance is laser intensity exposure in the fibre core during the FBG inscription, as shown by comparing the fatigue results for the Type I and Type II FBGs. Writing with a laser pulse peak intensity above the damage threshold significantly reduces the tensile fatigue strength of the inscribed Type II gratings. The differences in failure strain results between sample sets in Table 4 are possibly attributed to slight variations in the exposure parameters, such as laser fluence and an intensity level above the damage threshold. This will require further experimental validation.

### 3.1. Microscopic Inspection of the FBG Sensor Region

**Figure 4:**
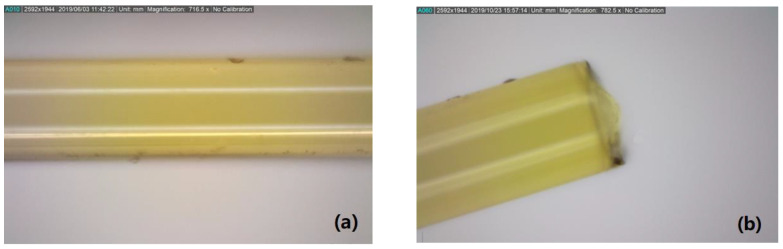
Representative microscope images at 716.5× magnification in the region of the sensor for a Type II trans-jacket FBG: (**a**) microscope image of the optical fibre at the grating, and (**b**) image of the failure location post-fatigue test.

The sensor region of all optical fibres was examined prior to fatigue testing using an optical light microscope to identify surface mechanical damage that could initiate premature sensor failure. Figure 4a shows an example 716.5× magnification micro-inspection of the fibre optic surface in the region of the Type II FBG sensor. All trans-jacket FBG sensors had a pristine surface. Figure 4b shows an example post-fatigue microscope inspection at the failure region of a Type II trans-jacket FBG sensor. Again, no surface mechanical damage was observed.

### 3.2. The Pre- and Post-Fatigue Reflection Spectra Analysis

**Figure 5:**
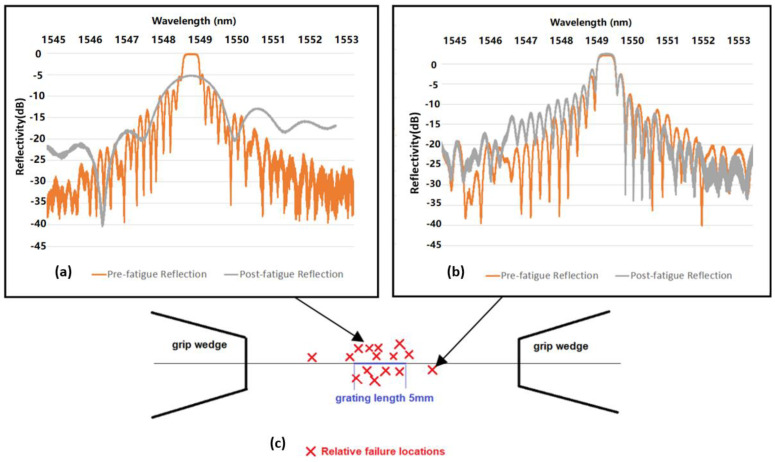
(**a**) Reflectivity spectra comparison of a Type II grating before and after it failed in the grating region. (**b**) Reflectivity spectra comparison of a Type II grating before and after it failed outside of the grating region. (**c**) Fibre fracture location schematic showing that Type II FBGs predominantly fracture in the grating region.

Figure 5c shows the approximate failure locations of one sample set of 15 Type II trans-jacket gratings. Failure location is determined by comparison of the reflective spectrum before and after fatigue failure together with microscope observations of the breakage location. Significant broadening of the post-fatigue reflective spectrum indicates a shorter FBG length, indicating the fractured fibre within the grating. The position in the grating can be estimated by the magnitude of the reflectivity drop, as shown in Figure 5a. In contrast, if a similar pre- and post-fatigue reflectivity profile is observed, then this is an indication that failure has occurred outside the grating, as shown in Figure 5b. Of the 80 Type II FBGs tested, approximately 85% fractured within the grating, and the remainder fractured adjacent to the grating (Figure 5c). This finding is consistent with the fact that the Type II inscription process causes physical damage in the fibre core, thus resulting in reduced mechanical robustness.

## 4. Conclusions

1. Type I trans-jacket FBGs written using fs-IR laser with active core alignment have high cyclic failure strain (above 36,000 µε) and fatigue life. Although Type II trans-jacket FBGs have a lower mean failure strain of approximately 17,000 µε, they can withstand more than one million cyclic load applications at 15,000 µε. Since aerospace structures seldom experience strains above 10,000 µε, even under extreme operational conditions, Type II trans-jacket FBGs written using the customised fs-IR grating inscription technique [40] have strong potential for long-term strain monitoring applications in aircraft and other high value engineering structures, particularly in high temperature environments where Type I gratings might be prone to erasure.
2. Type II trans-jacket FBGs are prone to breaking at the grating under quasi-static and cyclic loading, confirming the hypothesis that the damage inscription process creates weakness in the optical fibres leading to degradation of their fatigue performance.
3. The photosensitisation approach and fibre geometry had no significant impact on the mechanical fatigue performance of Type I and Type II gratings.

Future work will investigate high strain, fatigue-resistant adhesives suitable for broad area surface attachment of trans-jacket FBGs. This work supports a more extensive research project investigating the reliability of FBG sensors for structural health monitoring of defence and civil aerospace platforms as a potential replacement for electrical resistance foil strain gauges.

## Figures and Tables

**Table 1:** Optical fibre specifications.

Optical Fibre	Deuterium Loaded	Core (Clad) Diameter, µm	Numerical Aperture (Nominal)	Germanium Dopant
OFS: ClearLite POLY (4.6/125, 0.21NA)	Y&N	4.6 (125 ± 2)	0.21	~10 mol%
OFS: ClearLite POLY (8.4/125, 0.11NA)	Y&N	8.4 (125 ± 2)	0.11	~3 mol%
Fibercore: SM1500 (4.2/80)P	Y&N	4.2 (80)	0.29–0.31	~20 mol%
Fibercore: SM1500 (9/125)P	Y&N	9 (80)	0.29–0.31	~3 mol%

**Table 2:** Loading regime for Type I and Type II gratings.

Type of Inscription	Type I				Type II			
Peak Strain (µε)	30,000	32,000	34,000	36,000	12,000	14,000	16,000	18,000
Load Cycles (million)	0.5	0.5	0.5	0.5	0.5	0.5	0.5	0.5

**Table 3:** Fatigue test results for the Type I trans-jacket gratings.

Fibre Tested	Deuterium Loaded	Specimens Tested	Fatigue Results
OFS BF04446, ClearLite POLY (8.4/125, 0.11NA)	Y	15	Survived 2 million load cycle applications without breaking
OFS BF04446, ClearLite POLY (8.4/125, 0.11NA)	N	15	Survived 2 million load cycle applications without breaking
OFS BF06160-02, ClearLite POLY (4.6/125, 0.21NA)	Y	5	Survived 2 million load cycle applications without breaking
OFS BF06160-02, ClearLite POLY (4.6/125, 0.21NA)	N	5	Survived 2 million load cycle applications without breaking
FibreCore: SM1500 (4.2/80)P	Y	15	Survived 2 million load cycle applications without breaking
FibreCore: SM1500 (4.2/80)P	N	15	Survived 2 million load cycle applications without breaking
Fibercore: SM1500 (9/125)P	Y	5	Survived 2 million load cycle applications without breaking
Fibercore: SM1500 (9/125)P	N	5	Survived 2 million load cycle applications without breaking

**Table 4:** Fatigue test results for the Type II trans-jacket gratings.

Sample Set	Fibre Type	Deuterium Loaded	Specimens Tested	Mean Failure Strain, µε	95% Confidence Interval
1	OFS BF04446, ClearLite POLY (8.4/125, 0.11NA)	Y	15	17,600	±324
2	OFS BF04446, ClearLite POLY (8.4/125, 0.11NA)	N	15	17,900	±400
3	OFS BF06160-02, ClearLite POLY (4.6/125, 0.21NA)	Y	5	17,600	±445
4	OFS BF06160-02, ClearLite POLY (4.6/125, 0.21NA)	N	5	16,200	±500
5	FibreCore: SM1500 (4.2/80)P	Y	15	17,300	±588
6	FibreCore: SM1500 (4.2/80)P	N	15	18,100	±480
7	Fibercore: SM1500 (9/125)P	Y	5	17,100	±473
8	Fibercore: SM1500 (9/125)P	N	5	16,400	±643

## Data Availability

The data presented in this study are available on request from the corresponding author.
